# Presenting illness and mortality outcomes for patients intubated in an academic emergency department

**DOI:** 10.1186/2197-425X-3-S1-A667

**Published:** 2015-10-01

**Authors:** L DeLuca, T Durns, R Miller, Z Roward, A Pickering, J Yeaton, KR Denninghoff

**Affiliations:** University of Arizona, Department of Emergency Medicine, Tucson, United States

## Introduction

Patients who are intubated during the course of hospital care have a high mortality rate. 70% of critically ill Emergency Department (ED) patients are intubated in the ED or a prehospital, but little data describes mortality risk. Identification of mortality risk based on presenting illness would allow providers to improve both resuscitative efforts and advanced care planning.

## Objectives

To characterize the influence of presenting complaint on mortality for patients intubated in the ED.

## Methods

This study was performed in a Level I Trauma Center with an annual ED census of 77,000.

A cohort of patients intubated in the ED was identified and grouped into those who survived to discharge (SURVIVED), those who died after being designated DNR/DNI or having care withdrawn (WDCARE) per family wishes, and those who died after full resuscitative efforts (CODED). Patients were stratified into traumatic and medical complaints and mortality was compared between groups. Chart review characterized specific complaints and compared mortality for mechanisms of injury and organ system disorders.

## Results

**Figure 1:**
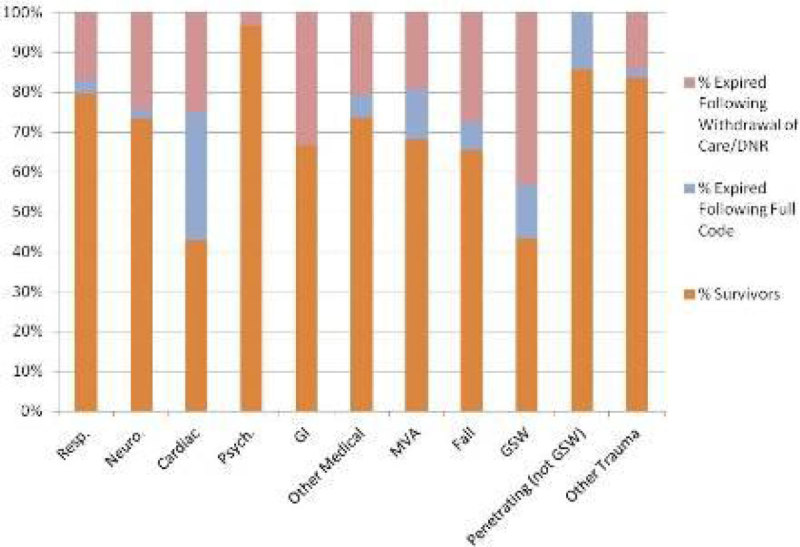
**Mortality by Presenting Complaint.**

533 patients were included. Overall 72.8% (388) SURVIVED, 7.5% (40) CODED and 19.7% (105) died after DNR or WDCARE. Of the 241 trauma patients, 10% (24) CODED and 21.2% (51) died after DNR or WDCARE. Of the 292 patients intubated after presenting with medical complaints, 5.5% (16) CODED and 18.5% (54) died after DNR or WDCARE. Cohorts were further divided by organ system disease and mechanism of injury (Table 1 & 2, Figure 1). For trauma patients, gunshot wounds (GSW) and falls had the highest mortality at 56.7% and 34.5%, respectively. For medical patients, 57% with cardiac complaints and 33% of gastrointestinal complaints expired. Psychiatric complaints had the lowest mortality at 3.5%.

**Table 1:** Medical Presenting Complaints.

	Respiratory	Neurologic	Cardiac	Psychiatric	GI	Other Medical
SURVIVED	47 (79.7%)	63 (73.3%)	12 (42.9%)	55 (96.5%)	6 (66.7%)	39 (73.6%)
CODED	2 (3.4%)	2 (2.3%)	9 (32.1%)	0 (0.0%)	0 (0.0%)	3 (5.7%)
WDCARE / DNR	10 (16.9%)	21 (24.4%)	7 (25.0%)	2 (3.5%)	3 (33.3%)	11 (20.8%)
TOTAL	59	86	28	57	9	53

**Table 2:** Trauma Presenting Complaints.

	MVA	Fall	GSW	Other Penetrating	Other Blunt
SURVIVED	81 (68.1%)	19 (65.5%)	13 (43.3%)	12 (85.7%)	41 (83.7%)
CODED	15 (12.6%)	2 (6.9%)	4 (13.3%)	2 (14.3%)	1 (2.0%)
WDCARE / DNR	23 (19.3%)	8 (27.6%)	13 (43.3%)	0 (0.0%)	7 (14.3%)
TOTAL	119	29	30	14	49

## Conclusions

Patients who were intubated in the ED for traumatic injuries had higher mortality rates than those intubated for medical reasons. GSW and cardiac patients had the highest rates of death among traumatic and medical presentations, respectively. GSW patients were also the most likely to expire following withdrawal of care. The ability to understand which patients are most at risk for mortality will help determine which ED patients need further investigation to allow for the development of risk assessment tools.
